# Non-enhanced magnetic resonance imaging-based radiomics model for the differentiation of pancreatic adenosquamous carcinoma from pancreatic ductal adenocarcinoma

**DOI:** 10.3389/fonc.2023.1108545

**Published:** 2023-01-23

**Authors:** Qi Li, Xuezhou Li, Wenbin Liu, Jieyu Yu, Yukun Chen, Mengmeng Zhu, Na Li, Fang Liu, Tiegong Wang, Xu Fang, Jing Li, Jianping Lu, Chengwei Shao, Yun Bian

**Affiliations:** ^1^ Department of Radiology, Changhai Hospital, Navy Medical University, Shanghai, China; ^2^ Department of Radiology, 96601 Military Hospital of PLA, Huangshan, Anhui, China

**Keywords:** pancreatic neoplasms, carcinoma, pancreatic ductal, carcinoma, adenosquamous, magnetic resonance imaging, diagnosis, differential

## Abstract

**Purpose:**

To evaluate the diagnostic performance of radiomics model based on fully automatic segmentation of pancreatic tumors from non-enhanced magnetic resonance imaging (MRI) for differentiating pancreatic adenosquamous carcinoma (PASC) from pancreatic ductal adenocarcinoma (PDAC).

**Materials and methods:**

In this retrospective study, patients with surgically resected histopathologically confirmed PASC and PDAC who underwent MRI scans between January 2011 and December 2020 were included in the study. Multivariable logistic regression analysis was conducted to develop a clinical and radiomics model based on non-enhanced T1-weighted and T2-weighted images. The model performances were determined based on their discrimination and clinical utility. Kaplan-Meier and log-rank tests were used for survival analysis.

**Results:**

A total of 510 consecutive patients including 387 patients (age: 61 ± 9 years; range: 28–86 years; 250 males) with PDAC and 123 patients (age: 62 ± 10 years; range: 36–84 years; 78 males) with PASC were included in the study. All patients were split into training (n=382) and validation (n=128) sets according to time. The radiomics model showed good discrimination in the validation (AUC, 0.87) set and outperformed the MRI model (validation set AUC, 0.80) and the ring-enhancement (validation set AUC, 0.74).

**Conclusions:**

The radiomics model based on non-enhanced MRI outperformed the MRI model and ring-enhancement to differentiate PASC from PDAC; it can, thus, provide important information for decision-making towards precise management and treatment of PASC.

## Introduction

Pancreatic adenosquamous carcinoma (PASC) and pancreatic ductal adenocarcinoma (PDAC) are two subtypes of pancreatic cancer (PC). PASC is rarer than PDAC, which accounts for only 1–4% of exocrine pancreatic malignancies (1). Pathologically, PASC is defined as PC with more than 30% malignant squamous cell carcinoma mixed with ductal adenocarcinoma (2). Recently, several studies have reported that adding cisplatin or oxaliplatin to traditional chemotherapy improves the overall survival of patients with PASC (3–5). Furthermore, increasing evidence indicates the sensitive potential of immunotherapy on PASC (5–7). However, the identical symptoms, manifestation (8), and similar imaging characteristics to PDAC pose challenges to preoperative discrimination of PASC from PDAC. At the moment, endoscopic ultrasonography-guided fine−needle aspiration is the only preoperative tool enabling tissue acquisition. However, it is an invasive, operator−dependent procedure that is unavailable in many centers (9). Therefore, there is an urgent need to non-invasively discriminate between PASC and PDAC.

Computed tomography (CT) and magnetic resonance imaging (MRI) are widely used for PC diagnosis and preoperative evaluation (10). Previous studies showed that the discrimination of PASC from PDAC using conventional imaging characteristics is helpful (11–14), especially in the ring-enhancement [ring-like enhancement with a relatively hypo-vascular central area on contrast-enhanced images (11)]. However, ring-enhancement was not ideal as it had a sensitivity of only 65.2% (11). Recently, CT radiomics has been used for diagnosing PASC and has shown good discrimination with an AUC of 0.98 using enhanced CT (15) and 0.80 using unenhanced CT (16). However, to the best of our knowledge, MRI radiomics models have not been reported to differentiate PASC from PDAC preoperatively (17). Moreover, the segmentation of pancreatic tumors of CT scans needed manual delineation, which was not only a laborious task but also a difficult task to achieve because of unavoidable interobserver variability in a previous study (18).

Therefore, this study aimed to develop and validate a fully automatic radiomics model (i.e., automatic rather than manual segmentation) from non-enhanced MRI to differentiate PASC from PDAC.

## Materials and methods

### Patients

**Figure 1:**
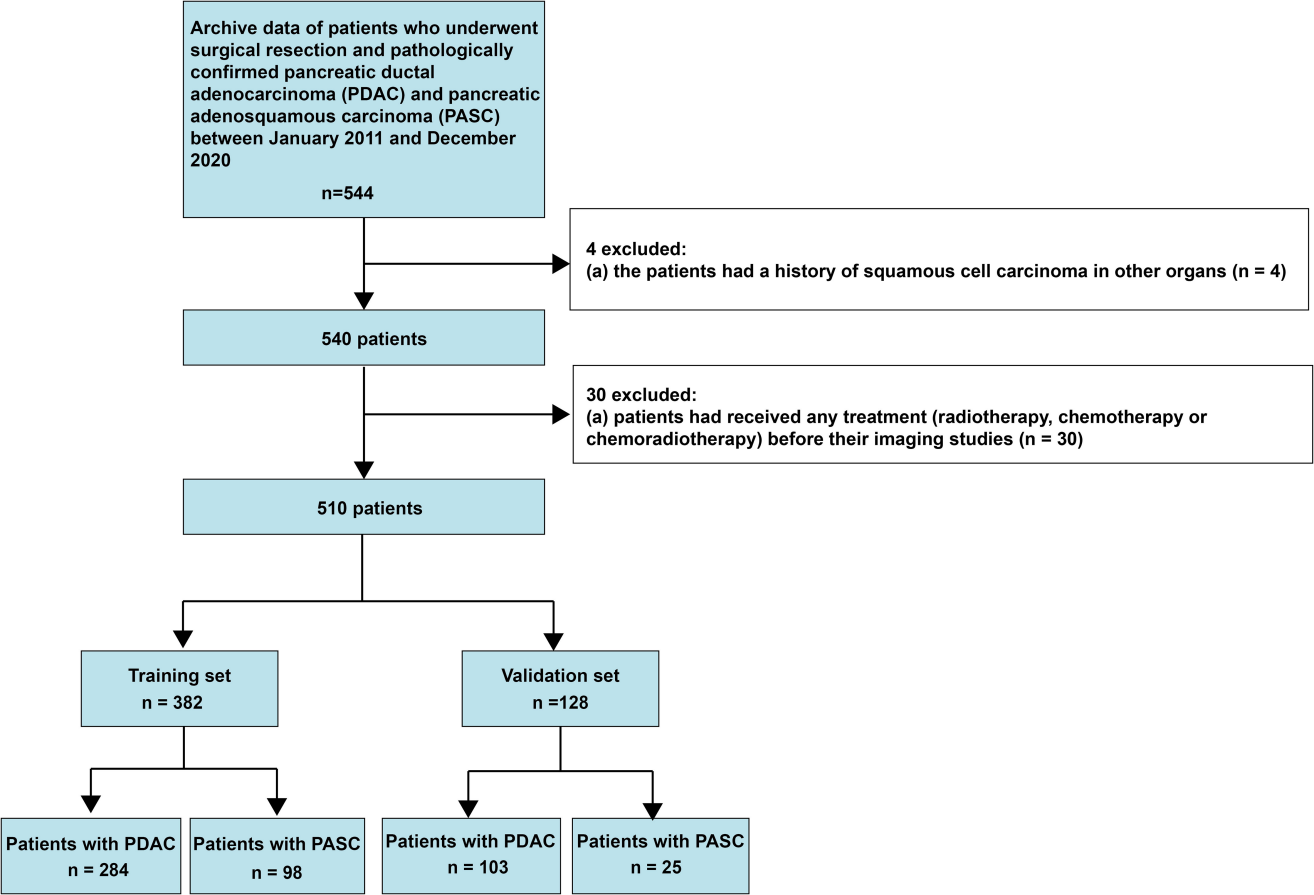
Flow chart illustrating patient selection process.

This retrospective study was approved by the Biomedical Research Ethics Committee of our institution, and the need to obtain informed consent from patients was waived. Data were collected on consecutive patients with histopathologically diagnosed PASC and PDAC between January 2011 and December 2020. A total of 544 patients diagnosed with PASC and PDAC met the inclusion criteria, which were (1): histopathologically confirmed PDAC and PASC and (2) having undergone enhanced MRI examination within 30 days of surgery. The exclusion criteria were as follows: (1) a history of squamous cell carcinoma in other organs and (2) having undergone neoadjuvant chemotherapy before imaging examination. The inclusion and exclusion criteria are presented in **Figure 1**. This study fundamentally followed the guidelines in the transparent reporting of a multivariable prediction model for individual prognosis or diagnosis (TRIPOD) consensus (19). It should be noted that this study included the patients who all underwent enhanced MRI, because the performance of ring-enhancement was compared with the radiomics model.

### MR protocols

All patients in this study underwent contrast-enhanced MRI of the pancreas using a 3.0-T system (Signa HDxt MR750 3.0-T, GE Healthcare, Milwaukee, USA; Skyra 3.0-T, Siemens, Erlangen, Germany). All patients were positioned supine, with a phased-array receiver coil covering the upper abdomen. The breath-hold single-shot fast-spin echo cross-sectional T2W sequence (repetition time/echo time [TR/TE], 6316/87 ms; field of view [FOV], 360 × 420 mm^2^; matrix, 224 × 270; slice thickness, 5 mm; slice gap, 1 mm) and unenhanced cross-sectional T1-weighted(T1W) fat-suppressed sequence (TR/TE = 2.58/1.18 ms; FOV = 440 × 440 mm^2^; matrix = 224 × 270; slice thickness = 5 mm; no slice gap) were used. Dynamic contrast-enhanced images, including the arterial phase (15 s), pancreatic parenchymal phase (20 s), and portal venous phase (40 s) images, were obtained using a fat-suppressed sequence (volumetric interpolated breath-hold examination; TR/TE=3.0/1.3 ms; FOV = 440 × 440 mm^2^; slice thickness = 5 mm; matrix = 224 × 270) after intravenous injection of gadopentetate dimeglumine (Magnevist and Gadovist, Bayer Schering Pharma, Berlin, Germany). A contrast agent dose of 0.2 mL/kg was administered intravenously at a flow rate of 2 mL/s, followed by 20 mL of normal saline (to flush the tube).

### Imaging analysis

Cross-sectional T1WI, T2WI and dynamic contrast-enhanced images were used for analysis. All images were analyzed by two abdominal radiologists (YB and QL, with 20 and 10 years of experience, respectively) blinded to the clinical and pathological details. Notably, the discordance between the two radiologists was resolved by consensus.

On the basis of a comprehensive literature research and our local experience on PASC and PDAC, the following characteristics were assessed (1): MRI-reported tumor size (the maximum cross-sectional diameter of the tumor (20)); (2) tumor location (head/uncinate or body/tail (21)); (3) pancreatitis (peripancreatic fluid (22)); (4) diameter of main pancreatic duct (MPD); (5) common bile duct (CBD) cutoff and dilation (> 10 mm (23)); (6) cyst (presence of pseudocysts and retention cysts with high signal intensity on T2-weighted (T2W) image (24)); (7) contour abnormality (bulging contours or loss of normal lobulation (25)); (8) parenchymal atrophy (anteroposterior body diameter less than 20 mm (23)); (9) MRI-reported lymph node (LN) metastasis (LN short-axis diameter > 10 mm (21)); and (10) ring-enhancement (26).

### Tumor annotation and segmentation

The MRIs of all patients were loaded into three-dimensional (3D) Slicer version 4.8.1 (an open-source software; https://www.slicer.org/), and regions of interest were manually drawn slice-by-slice on T1WI and T2WI (QL). A senior radiologist (YB) delineated all patients. We trained an automatic tumor segmentation model in the training set. Then this automatic segmentation model was applied in the validation set. The mask of the automatic segmentations was compared with the mask delineated by the senior radiologist using dice similarity coefficients (DSCs) in the validation set.

Moreover, 50 randomly selected patients including 25 patients with PDAC and 25 patients with PASC were used for delineation studies of pancreatic tumors (QL and WBL) for interobserver and intraobserver DSCs. Specifically, delineation was conducted two weeks apart in the intraobserver study.

The segmentation network of pancreatic tumors using nnU-Net, a robust adaptive framework based on 2D U-Net and 3D U-Net, was used for pixel-level semantic segmentation of NII data. In addition, nnU-Net automatically generated network structures and hyperparameters according to the characteristics of the data. The input patch size was 64 × 192 × 192, with a batch size of two. A total of five downsampling operations was performed, which resulted in a feature map size of 2 × 6 × 6 in the bottleneck. The initial number of convolutional kernels was set to 32, which was doubled with each down-sampling to a maximum of 320. The number of kernels in the decoder mirrors was in the encoder. Leaky rectified linear units (leaky ReLUs) were used as the nonlinear activation functions. Instance normalization was used for the feature map normalization. The training objective was the sum of the dice and cross-entropy losses.


$$\text{L}={\text{L}}_{\text{dice}}+{\text{L}}_{\text{ce}}$$

Where P is the predicted segmentation result, and T is the labeled segmentation result.


$$L_{dice}=\;1-\;\frac{2\left|P\wedge T\right|}{\left|P\right|+\left|T\right|}$$

y_i_ indicates the label value, and ${\text{y}}_{\text{i }}^{\prime }$ indicates the predictive value


$${\text{L}}_{\text{ce}}=-\sum_{\text{i}=1}^{\text{n}}{\text{y}}_{\text{i}}{\text{logy}}_{\text{i}}^{\prime }$$

Loss operated in one class, which labels the pancreatic tumor. The nnU-Net used stochastic gradient descent with an initial learning rate of 0.01 and a Nesterov momentum of 0.99. Training runs were conducted for 1000 epochs, where one epoch was defined as 250 iterations. The learning rate decayed using a polynomial schedule. Subsequently, training patches were cropped from randomly selected training cases.

The network was fully trained using labeled MR images (382 for T1WI and T2WI). We tested the performance of the segmented network on the validation set (the same patients as the above-mentioned validation set). The nnU-Net is a free and open-source out-of-the-box segmentation tool, and the source code is publicly available on GitHub (https://github.com/MRCWirtz/nnUNet-1, accessed on April 12, 2022). The software requires only a set of annotated MRIs as input data and a mainstream computer with a powerful GPU.

### Radiomics feature extraction

#### Feature extraction

A total of three preprocessing steps were applied to the MRIs before the feature extraction. First, the image was resampled to 0.625 mm × 0.625 mm × 1 mm spacing to eliminate differences in revolution and slice thickness. Second, the bias field was corrected to compensate for inhomogeneous artifacts across the MRI volume by using low-pass bias filtering. Finally, intensity standardization was used to align the MRI signal intensity distributions across all the patient sets.

Next, radiomics features were extracted using the open-source Python package PyRadiomics on python3.7 (version 3.0). First, the processed images were converted into various transformed images using different methods, comprising square roots, squares, logarithms, gradients, and wavelet filters. Subsequently, the features of the first-order statistics, gray-level co-occurrence matrix, gray-level run-length matrix, gray-level size zone matrix, gray-level dependence matrix, and neighboring gray-tone difference matrix were extracted from each of the transformed images.

#### Feature and model selection

**Figure 2:**
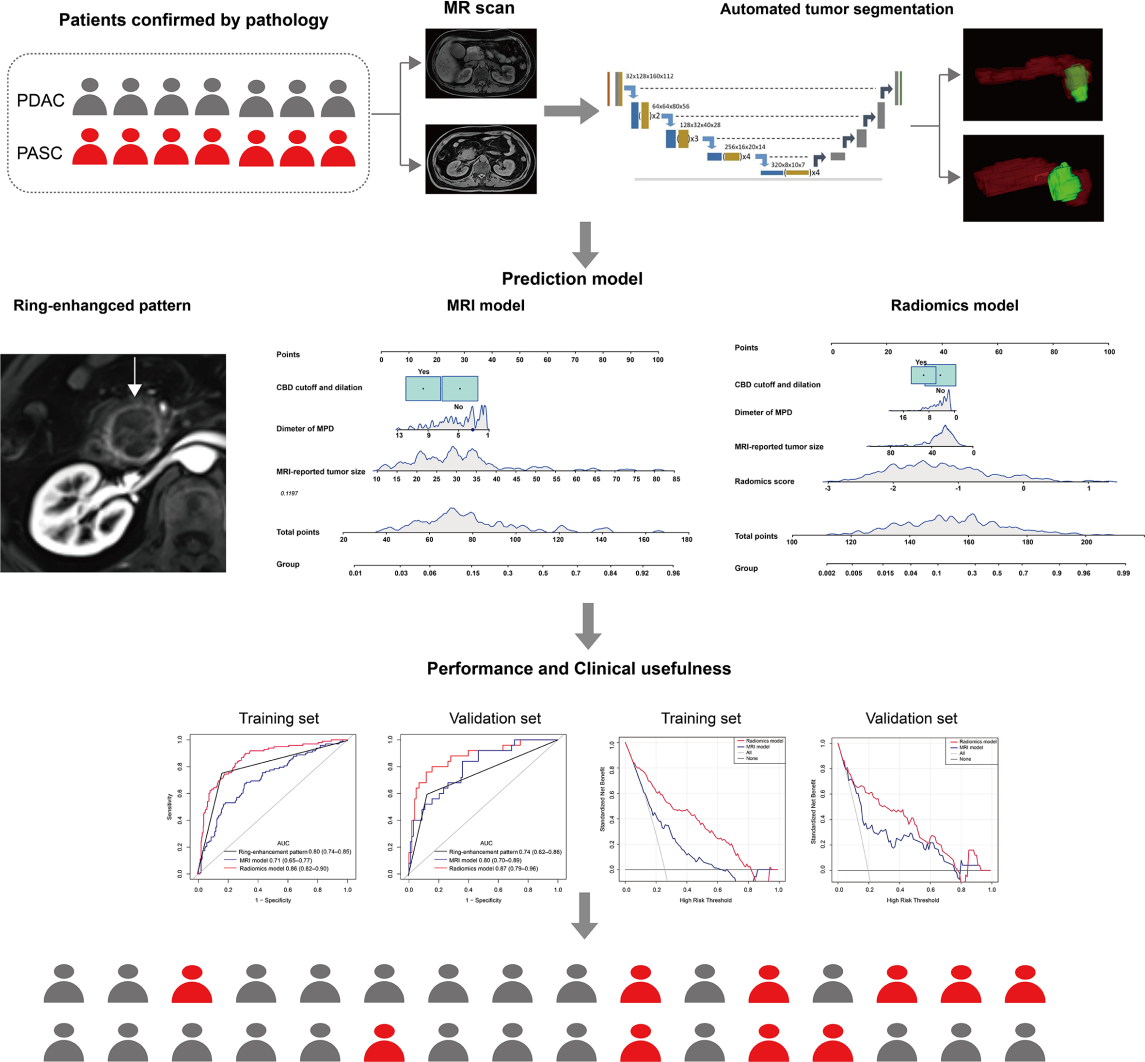
The workflow of the two models.

The following three feature selection steps reduced overfitting: variance analysis, Spearman correlation analysis, and the least absolute shrinkage and selection operator (LASSO) logistic regression algorithm, as have been demonstrated in previous radiomic studies (27). The sequential Bonferroni correction method was applied to adjust the baseline significance level (α = 0.05) for multiple testing biases (28, 29). Finally, a radiomics score (rad-score) was calculated for each patient using a linear combination of selected features weighted by their respective coefficients. **Figure 2** shows the flow diagram of this study.

### Statistical analysis

Normal distribution and variance homogeneity tests were performed for all the continuous variables. The Student’s t-test (normal distribution), Kruskal–Wallis H test (skewed distribution), and chi-square test (categorical variables) were used to compare the differences between the two groups. The interobserver DSCs between the automatic segmentation and manual delineation of tumors were reported. Interobserver agreement was quantified using the k statistic for categorical variables, which was graded as 0–0.20, poor agreement; 0.21–0.40, fair agreement; 0.41–0.60, moderate agreement; 0.61–0.80, good agreement; and 0.81–1.00, excellent agreement, and the intraclass correlation coefficient (ICC) for continuous variables, which was graded as 0–0.49, poor agreement; 0.50–0.75, moderate agreement; 0.76–0.90, good agreement; 0.91–1.00, excellent agreement. Deaths were defined as events, and deaths attributed to other causes were set as censored observations. Overall survival (OS) was calculated from the surgery date to the time of death or the end of the follow-up period.

Furthermore, univariate logistic regression analysis was used to select the significant clinical and radiomic characteristics (p< 0.05). A multivariable logistic regression analysis was conducted to develop a model for the differential diagnosis of PASC and PDAC, and a nomogram was constructed. We constructed a multivariable model using a stepwise regression method based on the Akaike information criterion to determine the best-fitting model (30). Subsequently, the discrimination of the model was evaluated using a receiver operating characteristic (ROC) curve, and the area under the ROC curve (AUC) was calculated concurrently. ROC curves were compared using the DeLong test. Additionally, the sensitivity, specificity and accuracy of the models were reported.

In this study, we developed two prediction models as follows: MRI and radiomics models. The MRI model was developed using conventional MRI characteristics, whereas the radiomics model was developed using conventional MRI characteristics and rad-score. The clinical usefulness of the models was tested using decision curve analysis (DCA) by quantifying the net benefit at different threshold probabilities. Finally, Kaplan–Meier estimates were applied to graph the survival curves and the log-rank test was used to analyze the differences between the predicted PASC group and the predicted PDAC group.

Statistical significance was defined as a two-tailed p-value< 0.05. All analyses were performed using the R software (version 3.3.3, R Foundation for Statistical Computing).

## Results

### Clinical and MRI characteristics

A tatal of 510 patinets were included in this study. Of the 510 included patients, 387 consecutive patients (age: 61 ± 9 years; range: 28–86 years; 250 males) had PDAC and 123 consecutive patients (age: 62 ± 10 years; range: 36–84 years; 78 males) had PASC. All patients were divided into training and validation sets according TRIPOD guidelines (19). The prediction model was developed on the training set of 382 consecutive patients (age: 61 ± 9.9 years; range: 28–85 years; 239 males), enrolled between January 2011 and February 2018. Thus, the validation set consisted of 128 consecutive patients (age: 60 ± 10 years; range: 35–83 years; 89 males), enrolled between March 2018 and December 2020. Interobserver agreements between two radiologists for MRI characteristics were excellent, and k statistic ranged from 0.91 to 0.93. The interobserver ICCs of MRI-reported tumor size were also good, ranging from 0.82 to 0.88. Significant differences were observed in tumor location, MRI-reported tumor size, CBD cutoff and dilation, diameter of MPD, and ring-enhancement in the training and validation sets. The BMI and MRI reported that LN status was significantly different in the training set. **Table 1** presents the summary of patient characteristics.

**Table 1:** Baseline characteristics of patients with PDAC and PASC.

Characteristics	Training set			Validation set		
	PDAC (n=284)	PASC (n=98)	*P* value	PDAC (n=103)	PASC (n=25)	*P* value
Sex, *n* (%)			0.94			0.85
Male	178 (62.68)	61 (62.24)		72 (69.90)	17 (68.00)	
Female	106 (37.32)	37 (37.76)		31 (30.09)	8 (32.00)	
Age, years (mean±SD)	61.93±9.4	61.68±9.7	0.60	59.82±9.54	63.2±10.41	0.92
BMI, kg/m^2^ (mean±SD)	22.85±2.87	22.65±3.49	0.03	23.04±3.08	22.79±2.88	0.79
Tumor location, *n* (%)			0.001			< 0.001
Head and neck	187 (65.85)	46 (46.94)		81 (78.64)	10 (40.00)	
Body and tail	97 (34.15)	52 (53.06)		22 (22.36)	15 (60.00)	
MRI-reported tumor size, cm (mean±SD)	2.94±1.23	3.77±1.49	0.003	2.81±0.92	4.24±1.76	< 0.001
Pancreatitis, *n* (%)			0.74			0.61
No	271 (95.42)	92 (93.88)		93 (90.29)	24 (96.00)	
Yes	13 (4.58)	6 (6.12)		10 (9.71)	1 (4.00)	
Dimeter of MPD, cm (mean±SD)	0.47±0.28	0.37±0.26	0.006	0.52±0.28	0.34±0.21	0.047
CBD cutoff and dilation, *n* (%)			< 0.001			0.006
No	157 (55.28)	77 (78.57)		47 (45.63)	19 (76.00)	
Yes	127 (44.72)	21 (21.43)		56 (54.37)	6 (24.00)	
Cyst, *n* (%)			0.76			0.51
No	264 (92.96)	92 (93.88)		95 (92.23)	24 (96.00)	
Yes	20 (7.04)	6 (6.12)		8 (7.77)	1 (4.00)	
Contour abnormity, *n* (%)			0.32			0.95
No	50 (17.61)	13 (13.27)		17 (16.50)	4 (16.00)	
Yes	234 (82.39)	85 (86.73)		86 (83.50)	21 (84.00)	
Parenchymal atrophy, *n* (%)			0.40			0.099
No	128 (40.07)	49 (50.00)		47 (45.63)	16 (64.00)	
Yes	156 (54.93)	49 (50.00)		56 (54.37)	9 (36.00)	
MRI-reported LN status, *n* (%)			0.017			0.38
No	158 (55.63)	68 (69.39)		56 (54.37)	16 (64)	
Yes	126 (44.37)	30 (30.61)		47 (45.63)	9 (36)	
Ring-enhancement pattern			< 0.001			< 0.001
No	238 (83.80)	24 (24.49)		90 (87.38)	10 (40.00)	
Yes	46 (16.20)	74 (75.51)		13 (12.62)	15 (60.00)	

PDAC, pancreatic ductal adenocarcinoma; PASC, pancreatic adenosquamous carcinoma; SD, standard deviation; BMI, body mass index; MPD, main pancreatic duct; CBD, common bile duct; LN, lymph node.

### Agreement between manual and automatic segmentation

The intraobserver and interobserver DSCs for manual segmentation were both moderate, ranging from 0.73 to 0.80 and 0.71 to 0.76, respectively, in the T1WI and T2WI sequences. Additionally, the DSCs between the manual segmentation and automatic segmentation were moderate, ranging from 0.77 to 0.81 in the two sequences for the validation set.

### Radiomics analysis

**Figure 3:**
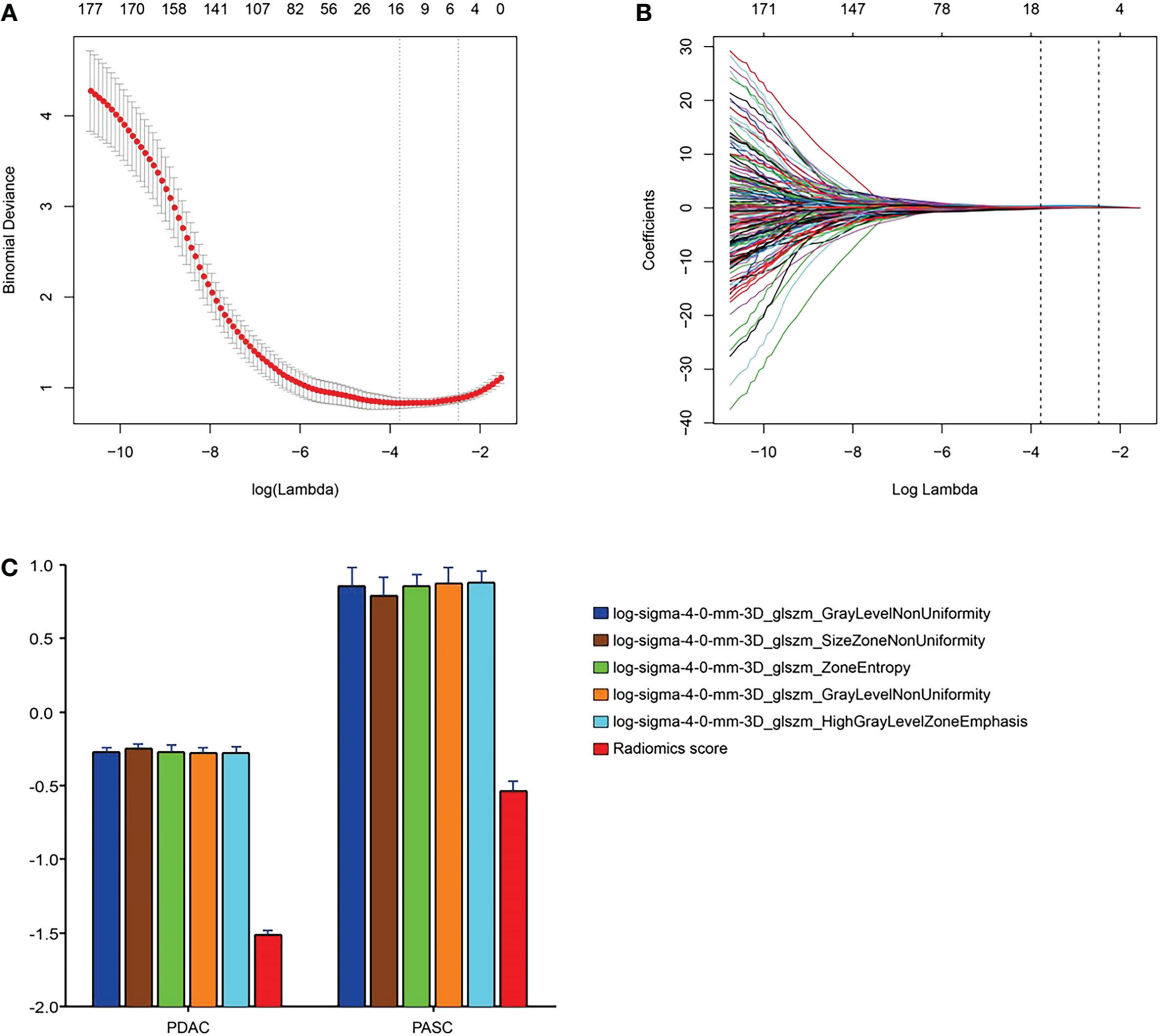
Radiomics feature selection using the parametric method, the least absolute shrinkage and selection operator (LASSO). **(A)** Selection of the tuning parameter (λ) in the LASSO model *via* 10-fold cross-validation based on minimum criteria. Binomial deviances from the LASSO regression cross-validation procedure were plotted as a function of log(λ). The y-axis indicates binomial deviances. The lower x-axis indicates the log(λ). Numbers along the upper x-axis represent the average number of predictors. Red dots indicate the average deviance values for each model with a given λ, and vertical bars through the red dots show the upper and lower values of the deviances. The vertical black lines define the optimal values of λ, where the model provides its best fit for the data. The optimal λ value of 0.08 with log(λ) = -2.48 was selected. **(B)** LASSO coefficient profiles of the 328 features. The dotted vertical line was plotted at the selected value using 10-fold cross-validation. The five resulting features with nonzero coefficients are indicated in the plot. **(C)** The error-bar chart of the five radiomics features and radiomics score.

We excluded radiomics features with non-significant differences between the groups or non-significant correlations between the two types of PC. Subsequently, the radiomics features were reduced to 328. Finally, the radiomics features were reduced to five by LASSO regression, and the LASSO formula was used to obtain the rad-score (**Table 2**). **Figure 3** shows the LASSO results for optimal features.

**Table 2:** The radiomics features selected by the least absolute shrinkage and selection operator logistic regression algorithm.

β	Radiomics features
0.11	log-sigma-4-0-mm-3D_glszm_GrayLevelNonUniformity (T1WI)
0.01	log-sigma-4-0-mm-3D_glszm_SizeZoneNonUniformity (T1WI)
0.30	log-sigma-4-0-mm-3D_glszm_ZoneEntropy (T1WI)
0.05	log-sigma-4-0-mm-3D_glszm_GrayLevelNonUniformity (T2WI)
0.39	log-sigma-4-0-mm-3D_glszm_ZoneEntropy (T2WI)

Radiomics score =-1.28+ 0.10701* log-sigma-4-0-mm-3D_glszm_GrayLevelNonUniformity (T1WI)

+ 0.01 × log-sigma-4-0-mm-3D_glszm_SizeZoneNonUniformity (T1WI)

+ 0.30 × log-sigma-4-0-mm-3D_glszm_ZoneEntropy (T1WI)

+0.05 × log-sigma-4-0-mm-3D_glszm_GrayLevelNonUniformity (T2WI)

+ 0.39 × log-sigma-4-0-mm-3D_glszm_ZoneEntropy (T2WI)

### Performance of ring-enhancement

The AUC, sensitivity, specificity, and accuracy of the ring-enhancement were 0.80 (95% confidence interval [CI]: 0.74–0.85), 75.51%, 83.80%, and 81.68%, respectively, in the training set and 0.74 (95% CI: 0.62–0.86), 60.00%, 87.38%, and 82.03%, respectively in the validation set.

### MRI model

Multivariable logistic regression analysis included MRI-reported tumor size, tumor location, diameter of MPD, and CBD cutoff and dilation. Consequently, MRI-reported tumor size (p*<* 0.001), diameter of MPD (p *=* 0.037), and CBD cutoff and dilation (p = 0.014) were selected for the MRI model (**Table 3**). In the MRI model, the AUC, sensitivity, specificity, and accuracy were 0.71 (95% CI: 0.65–0.77), 67.35%, 69.01%, and 68.59%, respectively, in the training set and 0.80 (95% CI: 0.70–0.89), 84.00%, 64.08%, and 67.97%, respectively, in the validation set.

**Table 3:** Multivariable logistic regression model distinguishing PASC from PDAC.

Variable	MRI model		Radiomics Model	
	OR (95% CI)	*P* value	OR (95% CI)	*P value*
Tumor size, mm	1.04 (1.02, 1.06)	< 0.001	0.96 (0.93, 0.99)	0.007
Dimeter of MPD, mm	0.90 (0.81, 0.99)	0.037	0.89 (0.79, 0.99)	0.035
CBD cutoff and dilation	0.49 (0.28, 0.86)	0.014	0.53 (0.27, 1.02)	0.057
Rad-score	NA	NA	12.04 (6.35, 22.80)	< 0.001

PDAC, pancreatic ductal adenocarcinoma; PASC, pancreatic adenosquamous carcinoma; MRI, magnetic resonance imaging; OR, odds ratio; MPD, main pancreatic duct; CBD, common bile duct; NA, not available.

### Radiomics model

**Figure 4:**
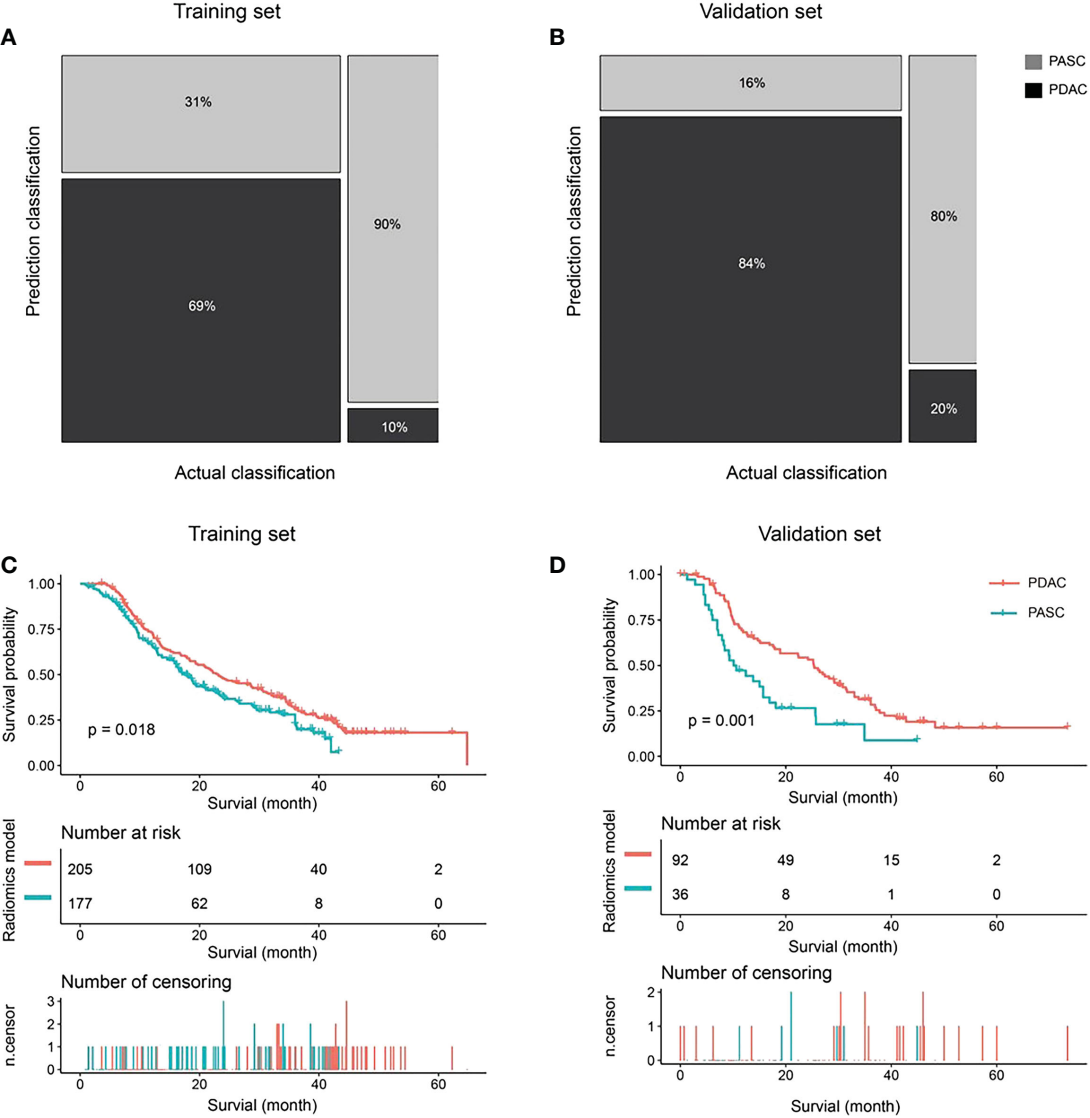
The classification and survival prediction of the radiomics model. **(A)** The mosaic plot of the training set. **(B)** The mosaic plot of the validation set. **(C)** The survival prediction of the radiomics model significantly shows longer survival for patients in the pancreatic ductal adenocarcinoma (PDAC) group than those in the pancreatic adenosquamous carcinoma (PASC) group in the training set. **(D)** The survival prediction of the radiomics model shows longer survival for patients in the PDAC group than those in the PASC group in the validation set significantly.

A total of 195 patients were accurately identified among 284 patients (69%, 195 of 284) in the PDAC group, whereas 88 patients (90%, 88 of 98) were accurately identified among 98 patients in the PASC group using the radiomics model in the training set (**Figure 4A**). In contrast, 87 patients were accurately predicted among 103 patients (84%, 87 of 103) in the PDAC group, and 20 patients (80%, 20 of 25) were accurately identified among 25 patients in the PASC group using the radiomics model in the validation set. (**Figure 4B**). The median follow-up duration was 18.7 months (IQR, 9.4–34.4 months) in the PDAC group and 13.7 months (IQR, 7.2–21.9 months) in the PASC group. During the observation period, 283 and 85 patients in the PDAC and 85 in the PASC groups died, respectively. A log-rank test revealed a longer survival duration in the predicted PDAC group than that in the predicted PASC group (training set: p = 0.018, validation set: p = 0.001) (**Figure 4C, D**).

**Figure 5:**
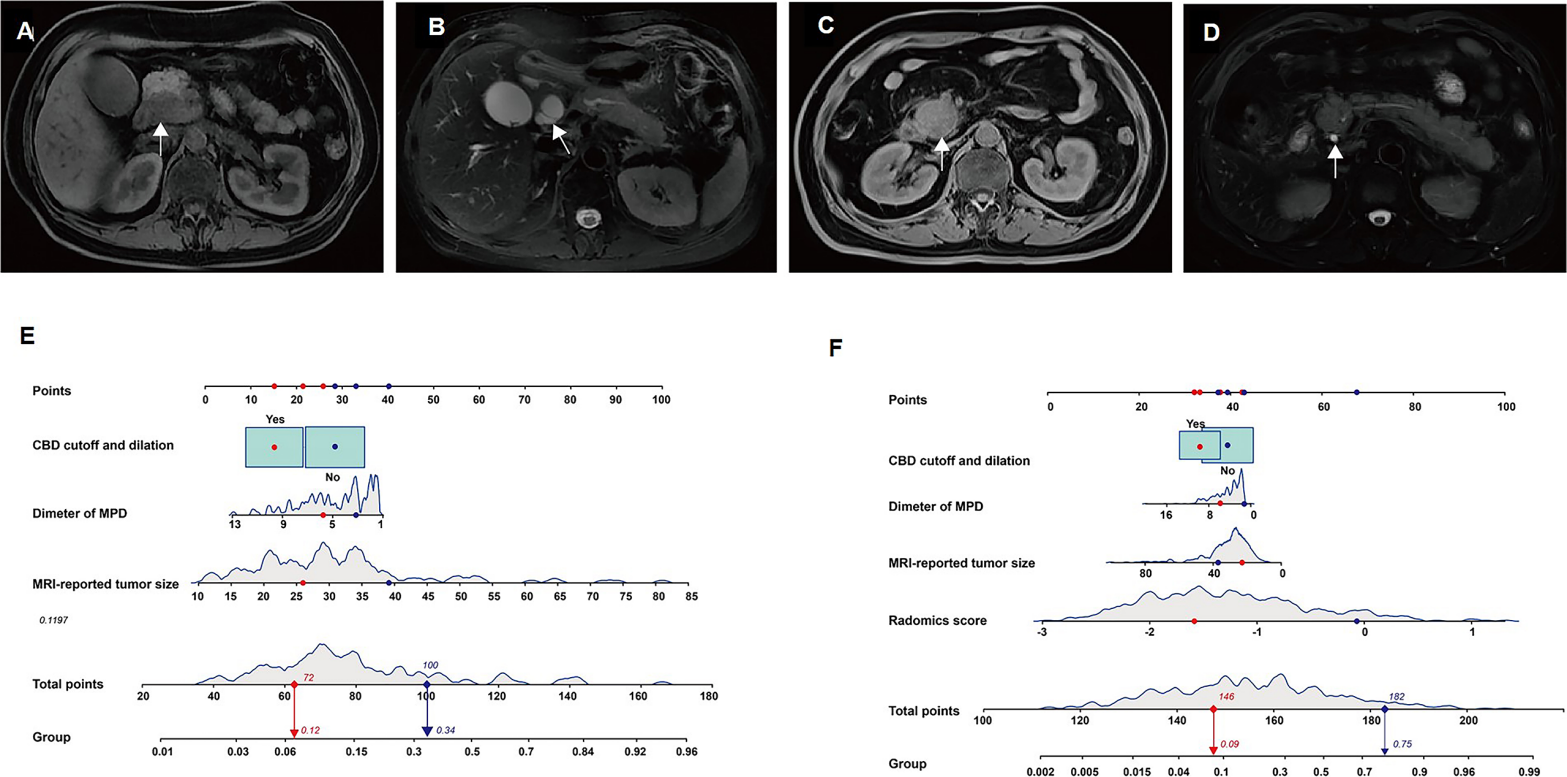
The two models accurately differentiate pancreatic adenosquamous carcinoma (PASC) from PDAC. Case 1: a 69-year-old woman with pathologically confirmed pancreatic ductal adenocarcinoma (PDAC). **(A)** Axial T1W image shows a hypoattenuated mass with an MRI-reported tumor size of 27 mm located at the pancreatic head (white arrow). **(B)**. Axial T2W image shows common bile duct (CBD) cutoff and dilation (white arrow). Case 2: a 54-year-old man with pathologically confirmed PASC. **(C)** Axial T1W image shows a hypoattenuated mass with an MRI-reported tumor size of 39 mm located at the pancreatic head (white arrow). **(D)**. The axial T2W image shows the typical CBD (white arrow). **(E)** According to the MRI model, the prediction probabilities of PASC are 0.12 (red arrow) and 0.34 (blue arrow) in cases 1 and 2, respectively. **(F)** According to the radiomics model, the prediction probabilities of PASC are 0.09 (red arrow) and 0.75 (blue arrow) in cases 1 and 2, respectively.

**Figure 6:**
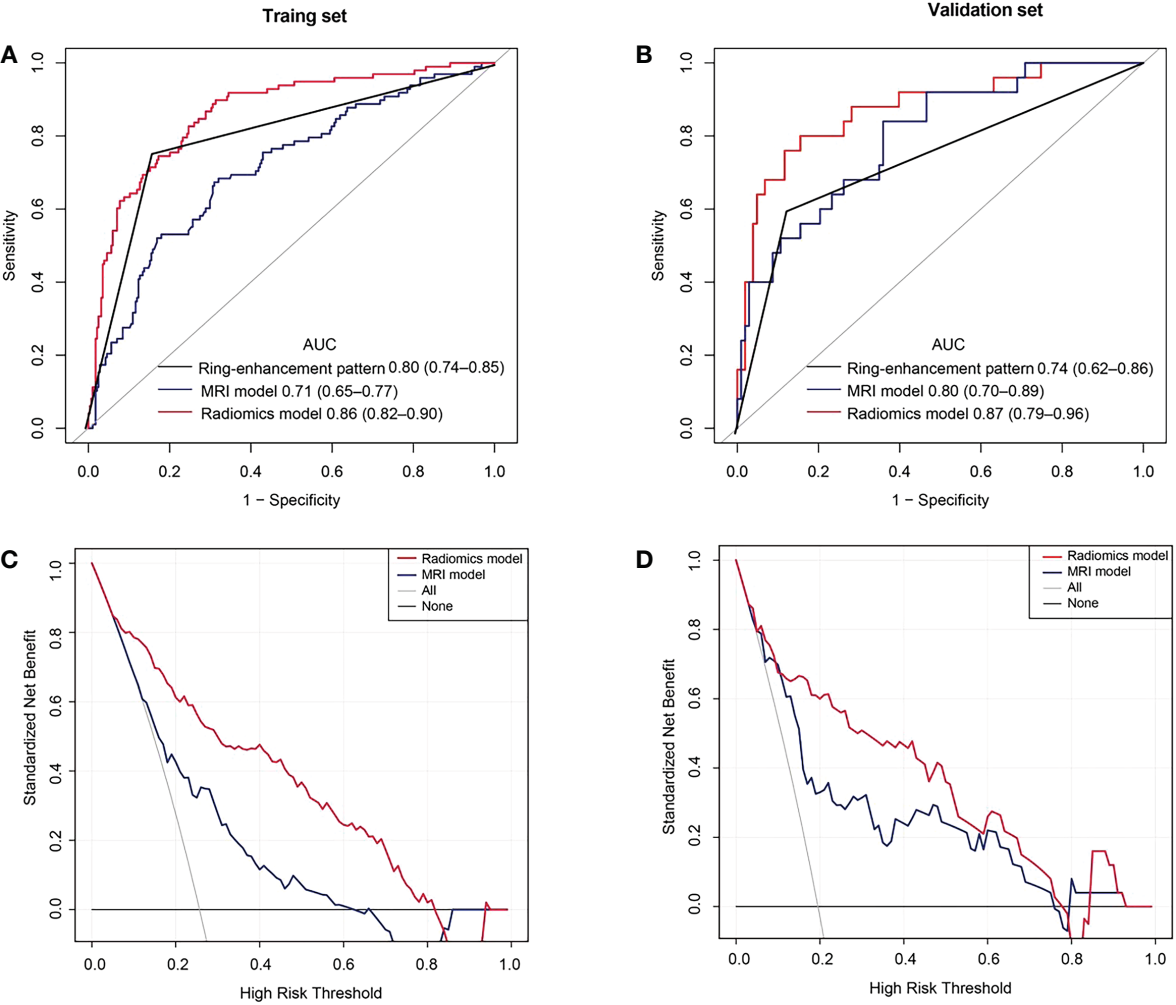
Receiver operating characteristic (ROC) and decision curve analysis (DCA) curves. **(A)** ROC curves in the training set. **(B)** ROC curves of the validation set. **(C)** The DCA curves of the training set. The y-axis represents the net benefit. The red line represents the radiomics model. The blue line represents the MRI model. The black line represents the hypothesis that all patients had pancreatic adenosquamous carcinoma (PASC). The gray line represents the hypothesis that all patients had pancreatic ductal adenocarcinoma (PDAC). The decision curves show that with a threshold probability between 0.05 and 0.81, using the radiomics model to predict PASC in the training set added more benefit than the treat-all-patients as a PDAC scheme or the treat-none as a PASC scheme. **(D)** The DCA curves show that with a threshold probability between 0.02 and 0.78, using the radiomics model to predict PASC in the validation set provided more benefits than the treat-all-patients as a PDAC scheme or the treat-none as a PASC scheme.

Furthermore, the multivariable logistic regression analysis included MRI-reported tumor size, tumor location, MPD diameter, CBD cutoff and dilation, and rad-score. Consequently, MRI-reported tumor size (p = 0.007), diameter of MPD (p = 0.035), CBD cutoff and dilation (p = 0.057), and rad-score (p*<* 0.0001) were selected for the radiomics model. In the radiomics model, the AUC, sensitivity, specificity, and accuracy were 0.86 (95% CI: 0.82–0.90), 89.80%, 68.66%, and 74.08% in the training set and 0.87 (95% CI: 0.79–0.96), 80.00%, 84.47%, and 83.59% in the validation set (**Figure 4A, B**). There was a significant difference in AUCs between the MRI model and radiomics model (p< 0.001) according to the DeLong test in the training and validation sets. The results of the two multivariable logistic regression models are shown in **Table 3**. **Figure 5** shows the two cases using the two nomograms. The performances of all prediction models are shown in **Table 4** and **Figure 6A, B**.

**Table 4:** The Performance of the Prediction Models.

Performance	The ring-enhancement		The MRI model		The radiomics model	
	Training set	Validation set	Training set	Validation set	Training set	Validation set
AUC*	0.80(0.74,0.85)	0.74(0.62,0.86)	0.71(0.65,0.77)	0.80(0.70,0.89)	0.86(0.82,0.90)	0.87(0.79,0.96)
Sensitivity (%) ^†^	75.51	60.00	67.35	84.00	89.80	80.00
Specificity (%) ^†^	83.80	87.38	69.01	64.08	68.66	84.47
Accuracy (%) ^†^	81.67	82.03	68.59	67.97	74.08	83.59

Performance is presented as the area under the receiver operating characteristic curve (AUC), with 95% CIs and sensitivity and specificity values according to the optimal selected cutoff.

*Data in parentheses are AUCs, with 95% CIs in brackets.

†Data in parentheses are numbers of participants.

### Clinical utility

The DCA showed that if the threshold probability were 0.05–0.81 and 0.02–0.78 in the training and the validation sets, respectively, using the radiomics model to distinguish PASC from PDAC was more beneficial than the treat-all-patients as a PASC scheme or the treat-all-patients as a PDAC scheme (**Figure 6C, D**).

## Discussion

To our knowledge, this is the first study to combine conventional MRI characteristics and radiomic features using non-enhanced MRI to develop a prediction model for the discrimination of PASC from PDAC in patients with PC. Additionally, in the present study, the fully automatic segmentation of pancreatic tumors was more in line with real-world clinical application scenarios.

Previous studies have summarized the radiologic characteristics distinguishing PASC from PDAC, including ring-enhancement, exophytic tendency, ill-defined boundary, mild MPD dilatation, and large tumor size (11–13, 31). The most vital sign was the ring-enhancement, the diagnostic sensitivity, specificity for the diagnosis of PASC (65.2% and 89.6% respectively) (11). In this study, the diagnostic sensitivity, specificity of the ring-enhancement for the diagnosis of PASC were 75.51% and 83.80% for the training set, respectively, and 60.00% and 87.38% for the validation set, respectively. This result showed that the diagnostic performance of the ring-enhancement was not ideal. Furthermore, previous studies were based on contrast imaging, and the widespread use of gadolinium-based contrast agents has resulted in safety concerns, such as nephrogenic systemic fibrosis (32) and deposition and retention of gadolinium in the brain and other organs (33). Therefore, we only incorporated all the significant MRI characteristics from non-enhanced T1WI and T2WI to develop the MRI model. The MRI model yielded an AUC of 0.71 and 0.80 in the training and validation sets, respectively, indicating limited diagnostic efficiency. Given the limitations of diagnostic efficiency in differentiating PASC from PDAC, additional tools are required to solve this dilemma.

To improve diagnostic efficacy, we added radiomics signatures from non-enhanced T1WI and T2WI to the MRI model to develop the radiomics model. Radiomics, offering better disease characterization by extracting high-dimensional features beyond visual assessment (34), has been a promising method to evaluate the infiltration levels of CD8+ T-cells in patients with PDAC (35), the differential diagnosis of mass-forming pancreatitis from PDAC (36), and the grade of nonfunctioning pancreatic neuroendocrine tumors (37). Pathologically, the squamous component of PASC has been shown to express TP63, TP40, and CK5/6 and present with dense and eosinophilic cytoplasm, clear cell boundaries (38). Finally, five radiomics features from the gray-level size zone matrix (GLSZM) were selected for the radiomics model. As a second-order feature, GLSZM reflects the size and intensity of voxel clusters in a region of interest, which has proven useful when the main feature is heterogenous (35). The radiomics model outperformed the MRI model and yielded an AUC of 0.86 and 0.87 in the training and validation sets, respectively. Additionally, we observed that, in our radiomics model, the OS was significantly associated between the two types of patients predicted by the radiomics model. Therefore, the radiomics model improved the discrimination of PASC from PDAC, helping to improve clinical outcomes and predict the prognosis of patients.

Furthermore, the strength of this study was the fully automatic pipeline for pancreatic tumor segmentation using nnU-Net, which achieved a promising performance with satisfactory DSCs on T1WI and T2WI. Automatic segmentation of tumor has the potential to decrease interobserver and intraobserver inconsistencies and reduce the time taken for and labor involved in delineating tumors. In this study, the range of interobserver and intraobserver DSCs of the tumor were only between 0.71 and 0.80. These DSCs were lower than the DSCs of the tumor (DSCs, 0.77–0.81) between the automatic and manual segmentation, indicating that our automated segmentation model has a reproducibility similar to that of a radiologist. Finally, a fully automatic pipeline for pancreatic tumor segmentation is better suited to the real world than manual segmentation.

### Limitations

Our study had some limitations. First, this was a retrospective and single-center study with potential for bias. Second, an independent external validation dataset for another center will be needed in future study. Third, although our study only included sequences from non-enhanced MRI, the diffusion-weighted imaging and apparent diffusion coefficient should be investigated in the future to improve our model. Lastly, the postoperative information in many cases was unavailable, leaving potential room for bias, because patients were often followed up by the local oncologists.

## Conclusion

The radiomics model based on non-enhanced MRI outperformed the MRI model and ring-enhancement to differentiate PASC from PDAC and provided important decision-making information for precise management and treatment of PASC.

## Data availability statement

The original contributions presented in the study are included in the article/supplementary material. Further inquiries can be directed to the corresponding authors.

## Ethics statement

The studies involving human participants were reviewed and approved by Biomedical Research Ethics Committee of our institution of Changhai Hospital. Written informed consent for participation was not required for this study in accordance with the national legislation and the institutional requirements.

## Author contributions

Guarantors of the integrity of entire study: YB and CS. Study design or data acquisition or data analysis/interpretation: all authors. Manuscript drafting or manuscript revision: QL, XL and WL. Approval of final version of submitted manuscript: all authors. Literature research: JY, YC, MZ, FL and TW. Clinical studies: JL, XF and CS. Statistical analysis: YB. Manuscript editing: QL and XL. Supervision: YB and JPL All authors contributed to the article and approved the submitted version.
